# Longitudinal and transcultural assessment of the relationship between hallucinogens, well-being, and post-traumatic growth during the COVID-19 pandemic

**DOI:** 10.1038/s41598-023-41199-x

**Published:** 2023-09-11

**Authors:** José Carlos Bouso, Dóra Révész, Genís Ona, Giordano N. Rossi, Juliana M. Rocha, Rafael G. dos Santos, Jaime E. C. Hallak, Miguel Ángel Alcázar-Corcoles

**Affiliations:** 1 ICEERS-International Center for Ethnobotanical Education, Research, and Service, C/ Sepúlveda, 65 Bajos 2, 08015 Barcelona, Catalunya Spain; 2 Medical Anthropology Research Center (MARC), Universitat Rovira i Virgili, Tarragona, Catalunya Spain; 3 Department of Neurosciences and Behavior, Ribeirão Preto Medical School, University of São Paulo, Ribeirão Preto, Brazil; 4 National Institute of Science and Technology-Translational Medicine, São Paulo, Brazil; 5 Department of Biological and Health Psychology, School of Psychology, Autonomous University of Madrid (UAM), Madrid, Spain

**Keywords:** Risk factors, Psychology and behaviour

## Abstract

The COVID-19 pandemic has had a devastating impact on the health and wellbeing of the global population. This paper presents the results of a longitudinal transcultural study that was begun at the peak of the pandemic (in April, 2020). An online survey was used to collect data from English-, Spanish-, and Portuguese-speaking participants. The survey collected information about sociodemographics, lifestyle activities, COVID-19-related circumstances, and drug use (with an emphasis on hallucinogenic drugs), as well as involving psychometric questionnaires. Users of hallucinogenic drugs had higher psychological well-being and lower scores on psychopathology scales, both at baseline and during follow-ups. This difference was larger when users were distinguished by frequency of use, as regular users scored higher on psychological well-being and lower on psychopathology scales. Subjects with more psychological distress had lower scores for all scales of post-traumatic growth, but if they were regular hallucinogens users, they had higher scores for post-traumatic growth. When comparing the results between cultural contexts, heterogeneous results were obtained. There were more English-speaking regular users of hallucinogenic drugs. Further research should analyse the potential role of hallucinogens in large-scale catastrophes, with a special focus on post-traumatic growth.

## Introduction

The current public health concern brought about by the global COVID-19 crisis is more related with mental health rather than infectious diseases^1^. This is a consequence of the various challenging situations that populations around the globe are facing: the fear of the virus itself and the adverse health consequences if one is infected, the stigma experienced by certain groups that are in greater contact with the virus, the economic recession and unemployment, the bereavement of people who lost loved ones, and the lockdowns involving long-lasting social isolation, among other challenges.

Some countries duplicated the figures of mental health problems, and the numerous studies that are being published show increased anxiety, depression, post-traumatic stress disorder (PTSD), and general distress in the general population^2^. For instance, the Organization for Economic Cooperation and Development (OECD) recently reported that, since March 2020, the prevalence of anxiety and depression has increased and, in some countries, doubled. Additionally, the periods when the highest rates of mental distress were reported correlated with periods of marked increases in COVID-19 deaths and strict confinement measures^3^.

During the last decade, psychedelic drugs have been proposed as safe and efficacious tools for the treatment of mental disorders, including depression, anxiety, and PTSD^4,5^. There are at least two hallucinogens that are in Phase-III clinical trials for treatment-resistant depression and PTSD: psilocybin (the main alkaloid of *Psilocybe cubensis*) and 3,4-Methylenedioxymethamphetamine (MDMA), respectively. Certain countries have approved the use of these drugs in the framework of programs supporting compassionate use, including Canada, Israel, and the United States^6–9^. Both Australia and United States have allocated public funds to perform clinical trials with hallucinogens^10,11^. At the same time, a group of leading experts in the field of psychedelic research is recommending the use of such drugs to counter the mental health consequences of the pandemic^12,13^.

Many studies have been conducted on the effect of attending ceremonies involving psychoactive ethnobotanicals like ayahuasca on mental health^14,15^. Reported benefits relate to the grieving process^16,17^, emotional regulation^18^, mood^19^, drug use^20^ and general health^21,22^. Epidemiological studies about psychedelic use in the general population have also been performed, analysing government databases of health profiles of different populations, with results showing less domestic violence^23,24^, fewer suicide attempts^25–30^, and better general psychopathology^31,32^ among psychedelic users. According to the research, it can be hypothesized that psychedelic use may be a protective factor, rather than a risk factor, for mental health problems in the current situation, where a combination of pandemic-related factors threaten mental health^13,21^.

To date, few studies have been performed regarding the possible role of psychedelic use in preventing/treating mental health issues related to the COVID-19 pandemic. However, there is increasing interest in that regard^33^. Two retrospective observational studies have been published. The first one found that lifetime use of psychedelics was associated with better mental health during the COVID-19 pandemic^34^. The other one, a cross-sectional study performed by our group, further supported that finding^35^. Both studies were retrospective, so a direct causal relation could not be established. The current study includes the follow-ups with our previous sample^35^ at 2 and 6 months after baseline, aiming to provide a longitudinal assessment in order to determine whether better outcomes among regular users of psychedelics are stable over the long term.

## Materials and methods

This study was approved by the Research Ethics Committee of the Universidad Autónoma de Madrid (Autonomous University of Madrid, Spain). All experimental procedures were performed in accordance with the relevant guidelines and regulations, and all respondents provided informed consent.

### Sample

This longitudinal study recruited a total of 2971 subjects for the baseline assessment, 1024 subjects for the first follow-up performed two months later, and 455 subjects for the last follow-up, performed six months after the baseline assessment. All participants were asked to complete an online survey specifically developed for this study. The survey was originally developed in Spanish and then translated into Portuguese and English for international dissemination. The first assessment was launched while the populations of most Western countries were confined to their homes (April 7th, 2020), whereas distinct confinement measures were in place in different countries during both follow-ups. Through snowball sampling, researchers from various countries, including mainly Spain and Brazil, spread the questionnaires among direct contacts and through social media. The questionnaire was also shared on the websites of the Mental Health Post-graduate Program of the Ribeirão Preto Medical School at the University of São Paulo, in the scientific journal *Archives of Clinical Psychiatry*, and on websites offering information about psychedelics and cannabis (Lasdrogas.info, Cannabis Magazine, social media pages of ICEERS and local community websites). It was not mentioned in the instructions nor anywhere else in the survey that the study was investigating relationships between hallucinogenic drug use and other variables.

### Psychometric measures

The following validated questionnaires were included in the survey, as described previously in more detail^35^. First, the General Health Questionnaire (GHQ-12) was used to screen for psychological distress at all time points in its validated Spanish (α = 0.86–0.90)^36^, English (α = 0.82–0.86)^37^, and Portuguese (α = 0.83–0.86)^38^ versions. A higher score represents greater psychological distress.

We also used the Brief Symptom Inventory (BSI) in its validated English (α = 0.87–0.98)^39^, Portuguese (α = 0.73–0.88)^40^, and Spanish (α = 0.69–0.85)^41^ versions. The nine sub-scales (somatization, SOM; obsessive–compulsive, O–C; interpersonal sensitivity, IS; depression, DEP; anxiety, ANX; hostility, HOST; phobic anxiety, PHOB; paranoid ideation, PAR; and psychoticism, PSY) and the General Severity Index (GSI) were used, with higher scores indicating more severe symptoms. A T-score above 70 for each sub-scale and a score of 63 or above for the GSI indicate a clinical case.

Further, we used the Peritraumatic Stress Inventory (PSI) at baseline only. The PSI is a proposed measure of post-traumatic stress disorder (PTSD); that is, it measures symptoms associated with exposure to a potentially traumatic experience. The validated English version (α = 0.75–0.76)^42^ was used. The questionnaire was translated into Spanish and Portuguese by native Spanish and Portuguese researchers, respectively. Higher scores represent more intense symptoms resulting from exposure to potentially traumatic experiences.

Lastly, the Post-traumatic Growth Inventory (PGI) was also included in the two follow-up questionnaires, in its validated English (α = 0.90)^43^, Portuguese (α = 0.95)^44^, and Spanish (α = 0.95)^45^ versions. This provides a measure of positive changes perceived by the subject following a traumatic event. It contains five sub-scales (relationships, new possibilities, personal change, spiritual change, and life appreciation). Higher scores represent more post-traumatic growth (PTG).

### Hallucinogenic drug use

Participants were asked about their use of psychedelic drugs. We included the use of these psychedelic drugs: MDMA, ayahuasca, psilocybin-containing mushrooms, LSD, peyote, San Pedro, *Incilius alvarius* or 5-MeO-DMT, changa, and other psychedelics, as described previously^46^. Only at baseline, we categorized participants as (a) regular users (more than once per 6 months), (b) occasional users (tried it, but do not use it regularly), and (c) never-users. At the follow-ups, participants indicated whether they used each drug more often, less often, or the same as before.

At baseline, we also asked for the settings in which participants were using psychedelic drugs: alone, with friends or a partner, at parties or festivals, at rituals or in a therapeutic setting, or microdosing. We only added MDMA to the list of psychedelic drugs when it is used at rituals or in therapeutic settings. Furthermore, we assumed that this context remained the same throughout follow-up.”

### Other COVID-19 related indicators

We asked whether persons were diagnosed with COVID-19 or showed COVID-like symptoms, and whether they were still confined. We asked whether participants felt reluctant or afraid to leave their homes and about changes in their relationships, domestic conflicts, and employment/finances.

Additionally, subjects were asked about their lifestyle and the activities they were engaging in during confinement, including aerobic exercise; martial arts; music and singing; playing videogames; watching pornography; reading; watching TV, movies, series, or COVID-19 related news; weightlifting or bending; yoga; Pilates; and meditation. We also assessed changes in health habits since confinement.

We asked about medication use (with and without prescription), the doctors participants had seen during confinement, and whether they started any psychological therapy. We also asked them to rate their psychological well-being and their family environment during confinement on a scale from 1 to 10, with 1 indicating the worst and 10 the best score.

### Covariates

We recorded age, gender (male vs. female vs. others), and the language of the participant (Spanish, English, or Portuguese). For each participant, we recorded their partner status (yes/no). We asked for their religion (subsequently classified as atheist, agnostic, or religious) and whether they practice their religion. Each person was asked whether they had any physical or psychological conditions.

At each time point, we defined non-hallucinogenic drugs as alcohol, tobacco, cannabis, cocaine, amphetamines, and MDMA when not used in rituals or therapeutic settings.

### Statistical analysis

All variables were described as percentages or means and standard deviations. Sample characteristics are described at baseline, and at 2 and 6 months. We first did this analysis for the sociodemographic factors, religion, health factors, and substance use. Then we did the same for all COVID-19-related factors.

Next, in order to determine longitudinal associations between psychedelic drug users vs. non-users and the psychometric measures, we used generalized estimating equations (GEE) with an exchangeable correlation structure, which takes into account within-person correlations when examining multiple observations per subject and can handle missing values^46^. As dependent variables, we took these into account: (a) COVID-19-related items regarding psychological wellbeing, the home environment, and information given by politicians or the media, all items are unstandardized and rated on a scale of 1–10; (b) psychological distress (measured by the GHQ); (c) nine BSI scales and the GSI; and (d) five PTG scales. We performed the same analyses at baseline with regular vs. occasional vs. never users of psychedelic drugs as an independent variable, using the same set of psychometric measures as dependent variables. We corrected all these GEE models directly for age, gender, language of questionnaire, religion, practitioner of religion, and alcohol, tobacco, cannabis, cocaine, or amphetamine use, as described previously^35^.

Subsequently, we performed GEE analyses with peritraumatic stress, the GSI, psychological distress (GHQ), and occasional vs. never and regular vs. never users of psychedelic drugs as independent variables, and the five PTG scores as dependent variables. We corrected for age, gender, language of questionnaire, and religion. Cronbach’s Alpha was calculated for all the questionnaires used.

All analyses were conducted using SPSS version 24.0 (IBM Corp., Armonk, NY, USA). After Bonferroni-correction (p-value of 0.05 divided by 20), we set the significant p-value at 0.003, two-tailed.

## Results

### Reliability of questionnaires used

GHQ-12 questionnaire showed a Cronbach’s Alpha of 0.87, 0.89, and 0.89 for English, Portuguese, and Spanish versions, respectively. BSI questionnaire showed a Cronbach’s Alpha of 0.96, 0.97, and 0.96 for English, Portuguese, and Spanish versions, respectively. PSI questionnaire showed a Cronbach’s Alpha of 0.89, 0.82, and 0.85 for English, Portuguese, and Spanish versions, respectively. Lastly, PGI showed a Cronbach’s Alpha of 0.95, 0.94, and 0.95 for English, Portuguese, and Spanish versions, respectively.

### Sample characteristics and COVID-related items

Table 1 shows the sample characteristics at baseline, and at the 2 and 6-month follow-ups, while Table 2 shows COVID-related items at all time points. According to the epidemiological data, the number of participants who tested COVID-positive increased across the three assessments. Whereas 47% were confined at 2 months, 86% were confined at 6 months. The percentage of the sample in isolation increased as well, from 55.8% at 2 months to 74.1% at 6 months. Regarding personal income, nearly 50% of the sample experienced a reduction in the first weeks of the pandemic, while at 6 months only 33.4% of the sample indicated a reduction in income.

**Table 1:** Sample characteristics at baseline, and after 2 and 6 months.

	Baseline (N = 2971)	After 2 months (N = 1024)	After 6 months (N = 455)
Sociodemographics, N (%)			
Age (years, mean (SD))	36.3 (13.3)	–	–
Gender			
Men	852 (28.6)	303 (29.7)	149 (32.8)
Women	2087 (70.2)	714 (70.0)	305 (67.2)
Queer/Androgynous/others	18 (0.6)	3 (0.3)	0
Language questionnaire			
English^a^	671 (22.6)	204 (19.9)	0
Portuguese	691 (23.3)	179 (17.5)	125 (27.5)
Spanish	1609 (54.2)	641 (62.6)	330 (72.5)
Having a partner	1564 (52.6)	534 (52.2)	229 (50.3)
Religion, N (%)			
Religion groups			
Atheist	845 (28.4)	308 (31.9)	125 (29.3)
Agnostic	695 (23.4)	246 (25.5)	100 (23.5)
Religious	1279 (43.0)	411 (42.6)	201 (47.2)
Practitioner of religion	931 (31.3)	303 (34.3)	131 (33.0)
Health factors, N (%)			
Chronic diseases present	736 (24.7)	264 (25.8)	113 (24.8)
Mental diseases present	670 (22.5)	227 (22.2)	98 (21.5)
Substance use, N (%)			
Non-psychedelic			
Alcohol	1683 (56.6)	621 (60.6)	270 (59.3)
Tobacco	681 (22.9)	270 (26.4)	120 (26.4)
Cannabis	1212 (40.7)	303 (29.6)	106 (23.3)
Cocaine	331 (11.1)	44 (4.3)	23 (5.1)
Amphetamines	266 (8.9)	40 (3.9)	18 (4.0)
Psychedelic			
MDMA, ecstasy, molly^b^	701 (23.6)	101 (9.9)	47 (10.3)
Ayahuasca	609 (20.5)	186 (18.2)	70 (15.4)
Magic mushroom	841 (28.3)	208 (20.3)	64 (14.1)
LSD	737 (24.8)	156 (15.2)	48 (10.5)
Other psychedelics	487 (16.4)	119 (11.6)	37 (8.1)
Problems with drug use since covid	–	49 (4.8)	20 (4.4)
Context of use of psychedelic substances			
Not using anything	1899 (63.9)	–	–
Alone	65 (2.2)	–	–
With friends or partner	293 (9.9)	–	–
At parties or festivals	60 (2.0)	–	–
Rituals/therapeutic	315 (10.6)	–	–
Microdosing	5 (0.2)	–	–

^a^At follow-up 2, English speakers did not enter their email address, and we were unable to add them to the longitudinal analyses.

^b^Is only taken as a psychedelic drug when used for rituals or therapeutic settings.

**Table 2:** Factors relating covid-19 and the confinement period at baseline, and after 2 and 6 months.

COVID-19, N (%)	Baseline	After 2 months	After 6 months
Diagnosed with Covid-19			
No	2686 (90.4)	902 (88.1)	392 (86.2)
No test, but clear symptoms	275 (9.3)	112 (10.9)	38 (8.4)
Yes, tested positive	10 (0.3)	10 (1.0)	25 (5.5)
Confined	2878 (97.0)	481 (47.0)	389 (85.5)
Societal and economic changes, N (%)			
Fear to leave home	–	580 (56.7)	217 (47.7)
Changes in relationships			
No changes	–	372 (36.4)	82 (18.0)
I see less people	–	571 (55.8)	337 (74.1)
I see more people	–	80 (7.8)	36 (7.9)
Increased intradomestic conflicts	–	298 (29.1)	138 (30.3)
Lost job due to covid	595 (20.2)	145 (14.2)	42 (9.2)
Afraid to lose job next months	–	250 (24.4)	84 (18.5)
Decreased income due to covid	1404 (47.4)	420 (41.1)	152 (33.4)
Requested financial assistance	–	183 (17.9)	59 (13.0)
Received financial assistance		158 (30.2)	56 (29.9)
Financial difficulties due to Covid	–	207 (20.2)	78 (17.1)
Lifestyle during confinement, N (%)			
Aerobic exercise	1673 (56.3)	644 (63.1)	283 (62.2)
Covid-19 related news	2531 (85.2)	800 (78.4)	364 (80.0)
Martial arts	135 (4.5)	61 (6.0)	25 (5.5)
Music and singing	1026 (34.5)	390 (38.2)	167 (36.7)
Pornography	738 (24.8)	366 (35.8)	171 (37.6)
Reading	2436 (82.0)	913 (89.4)	421 (92.5)
TV, movies or series	2701 (90.9)	906 (88.7)	417 (91.6)
Videogames	1004 (33.8)	316 (31.0)	145 (31.9)
Weight lifting or bending	1114 (37.5)	375 (36.7)	148 (32.5)
Yoga, pilates or meditation	1683 (56.6)	397 (38.9)	148 (32.5)
Changes of health habits (eating, exercise, etc.)			
Better	–	346 (33.8)	126 (27.7)
Same	–	370 (36.1)	196 (43.1)
Worse	–	308 (30.1)	133 (29.2)
Mental and physical health, N (%)			
More medication with prescription	–	18 (1.8)	10 (2.2)
More medication without prescription	–	7 (0.7)	5 (1.1)
Doctor seen during confinement	–		
No doctor needed	–	665 (65.2)	220 (48.4)
Doctor treated me	–	230 (22.5)	181 (39.8)
Could not see my doctor	–	125 (12.3)	54 (11.9)
Psychological therapy started	–	98 (9.6)	56 (12.4)
Psychological wellbeing, mean (SD)	5.8 (2.1)	6.6 (1.9)	6.7 (1.9)
Family environment, mean (SD)	7.3 (2.2)	7.4 (2.0)	7.4 (2.0)
Worsened physical/mental condition	–	672 (65.6)	303 (66.6)

Various items are only recorded during the follow-up questionnaires, and at baseline they are not depicted.

A general increase in the frequency of engaging with different lifestyle activities during confinement can be observed, especially in terms of aerobic exercise, martial arts, music and singing, and reading. There was an increase in watching pornography as well. A general decrease was observed in the case of yoga, Pilates, and meditation.

**Figure 1:**
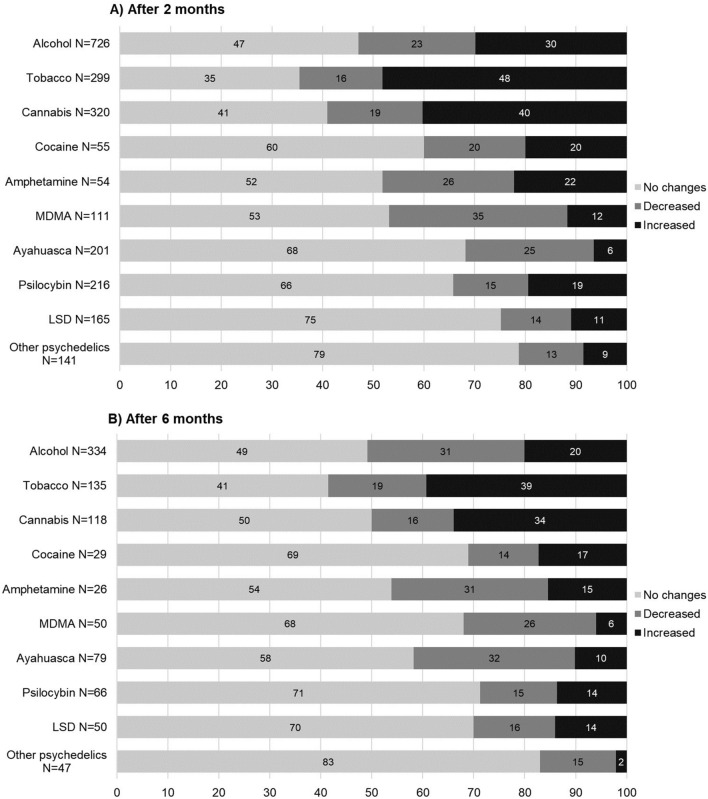
Effects of COVID-19 confinement on decreases, no change or increases in use of non-psychedelic and psychedelic drugs at both follow-ups, after 2 months (**A**) and 6 months (**B**).

As can be seen in Fig. 1, at the first follow-up, there was a general and marked increase in alcohol, tobacco, and cannabis use, while the most relevant drug use decrease occurred in the cases of amphetamines, MDMA, and ayahuasca. At the 6-month follow-up, the numbers were very similar, although the alcohol use did not increase as in the previous assessments.

After 2 months, persons with missing values were less often regular psychedelic users (14.9%) than in the persons with recorded data (20.1%), and the drop-outs scored higher on hostility (58.4 vs 57.2), and psychoticism (57.0 vs. 55.7). After 6 months, drop-outs scored lower on psychological well-being (5.8 vs. 5.9).

### Hallucinogenic drugs and psychometric measures

Among the sample, there was a general decrease in BSI scores across the follow-ups (see Supplementary Table 4), suggesting that stress peaked at the beginning of the pandemic and the associated lockdown measures. The use of hallucinogenic drugs over time was significantly associated with higher psychological wellbeing (B = 0.48; SE = 0.10; p < 0.001), and lower rates on the information given by the media (B = − 0.44; SE = 0.10; p < 0.001), as shown in Table 3. Hallucinogenic drug use was also associated with less psychological distress (B = − 0.73; SE = 0.16; p < 0.001), fewer obsessions or compulsions (B = − 1.82; SE = 0.40; p < 0.001), less depression (B = − 1.43; SE = 0.45; p = 0.001), less hostility (B = − 1.13; SE = 0.35; p = 0.001), less phobic anxiety (B = − 1.93; SE = 0.40; p = < 0.001), less paranoid ideation (B = − 1.24; SE = 0.36; p = 0.001), less psychoticism (B = − 1.02; SE = 0.32; p = 0.001), and lower GSI scores (B = − 0.76; SE = 0.22; p = 0.001). Regarding the PTG scores, psychedelic drug use was associated with higher scores on the new possibilities scale (B = 1.79; SE = 0.55; p = 0.001).

**Table 3:** Associations between longitudinally measured psychedelic drug users vs. non-users and baseline regular vs. occasional vs. never users of psychedelic drugs with psychometric measures.

	Longitudinal psychedelic users vs. non-users		Baseline occasional vs. never		Baseline regular vs. never	
	B (SE)	p	B (SE)	p	B (SE)	p
Covid-related items rated on 1–10 scale						
Psychological wellbeing	0.48 (0.10)	** < 0.001**	0.39 (0.12)	**0.001**	0.98 (0.12)	** < 0.001**
Home environment	0.05 (0.10)	0.60	− 0.08 (0.12)	0.53	0.25 (0.13)	0.06
Information given by politicians	− 0.22 (0.11)	0.04	− 0.28 (0.13)	0.04	− 0.40 (0.15)	0.01
Information given by media	− 0.44 (0.10)	< 0.**001**	− 0.37 (0.12)	**0.002**	− 0.66 (0.14)	** < 0.001**
Stress						
Psychological distress (GHQ)	− 0.73 (0.16)	** < 0.001**	− 0.11 (0.20)	0.58	− 0.65 (0.20)	**0.001**
Brief Symptom Inventory scores						
Somatization	− 0.99 (0.40)	0.01	− 0.46 (0.47)	0.32	− 2.31 (0.46)	** < 0.001**
Obsessive–compulsive	− 1.82 (0.40)	** < 0.001**	− 1.34 (0.49)	0.01	− 2.73 (0.50)	** < 0.001**
Interpersonal sensitivity	− 1.28 (0.44)	**0.004**	− 0.42 (0.52)	0.43	− 2.08 (0.52)	** < 0.001**
Depression	− 1.43 (0.45)	**0.001**	− 1.17 (0.55)	0.03	− 3.52 (0.53)	** < 0.001**
Anxiety	− 1.12 (0.42)	**0.01**	− 1.04 (0.48)	0.03	− 3.36 (0.49)	** < 0.001**
Hostility	− 1.13 (0.35)	**0.001**	− 0.22 (0.44)	0.62	− 1.65 (0.41)	** < 0.001**
Phobic anxiety	− 1.93 (0.40)	** < 0.001**	− 1.01 (0.48)	0.04	− 3.88 (0.48)	** < 0.001**
Paranoid ideation	− 1.24 (0.36)	**0.001**	− 0.81 (0.44)	0.06	− 2.93 (0.43)	** < 0.001**
Psychoticism	− 1.02 (0.32)0	**0.001**	− 0.14 (0.38)	0.72	− 1.30 (0.39)	**0.001**
General severity index	− 1.16 (0.29)	** < 0.001**	− 0.73 (0.35)	0.04	− 1.93 (0.35)	** < 0.001**
Posttraumatic growth scores^a^						
Relating to others	1.43 (0.70)	0.04	0.76 (0.83)	0.36	2.57 (0.94)	0.01
New possibilities	1.79 (0.55)	**0.001**	0.90 (0.62)	0.14	2.58 (0.68)	** < 0.001**
Personal strength	0.83 (0.50)	0.09	0.19 (0.56)	0.74	1.43 (0.61)	0.02
Spiritual change	0.65 (0.25)	0.01	0.17 (0.23)	0.45	0.91 (0.27)	**0.001**
Appreciation of life	0.48 (0.35)	0.16	0.07 (0.40)	0.86	1.00 (0.44)	.02

Generalized estimated equations were corrected for age, gender, language of questionnaire, religion, practitioner of religion, alcohol, tobacco, cannabis, cocaine or amphetamine use. Significant (Bonferroni-corrected) p-values ≤ 0.003 are represented bold.

^a^Posttraumatic growth was only recorded after 2 and 6 months, not at baseline.

When we examined the frequency of hallucinogenic drug use, regular users scored higher in terms of psychological wellbeing, and lower in terms of information given by the media and psychological distress. Regular users of hallucinogens had lower BSI scores, excluding GSI. Regular hallucinogenic drug use was associated with higher scores on the new possibilities and spiritual change scales of PTG.

### Stress, psychedelic drugs and posttraumatic growth

**Figure 2:**
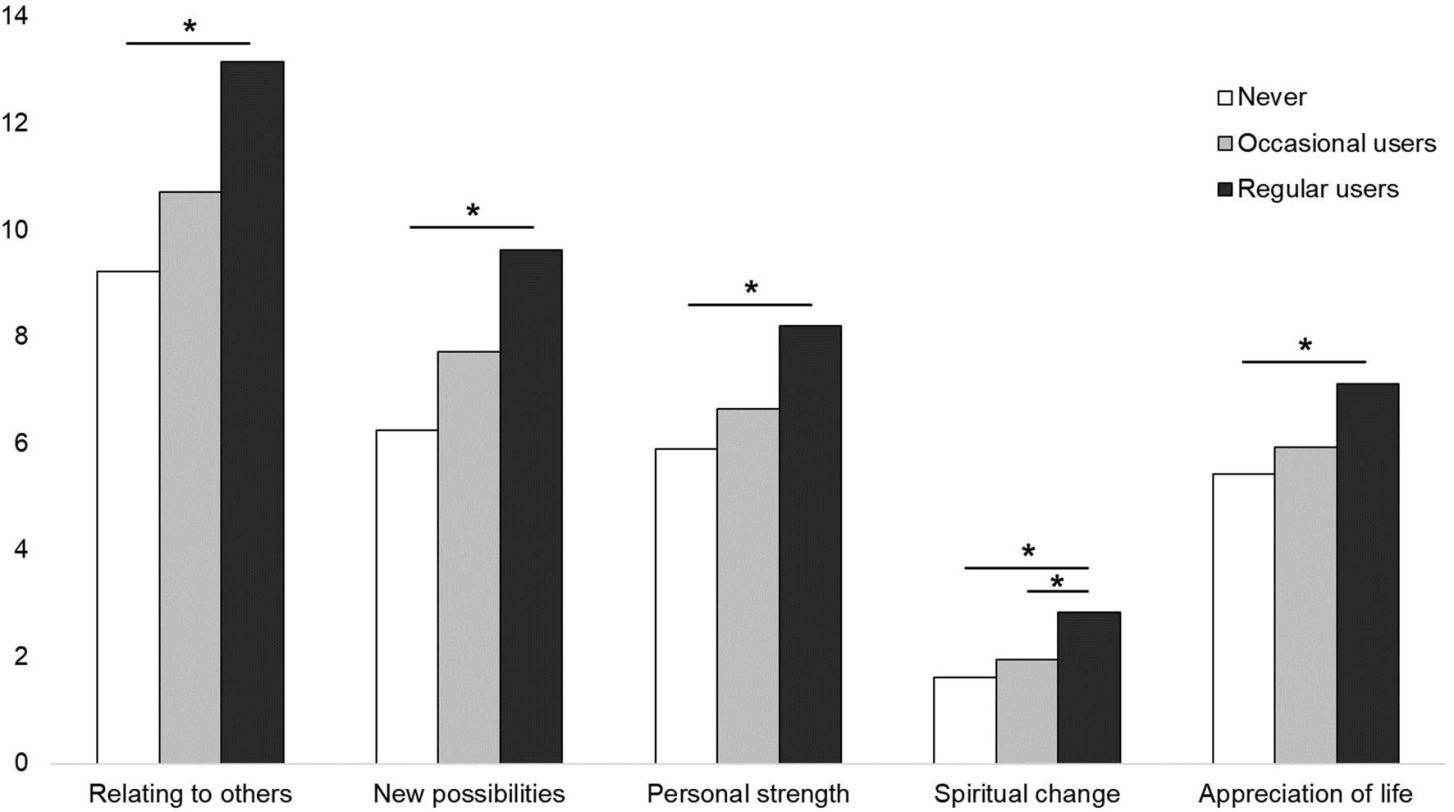
Means of five scales of posttraumatic growth among hallucinogenic drug users: never users (white) vs. occasional users (grey) vs. regular users (black), while correcting for age, gender, language of questionnaire, religion, peritraumatic stress score, general severity index, and psychological distress scores.

Table 4 shows the longitudinal analyses between various psychometric measures, psychedelic drugs, and the five scales of PTG. When entered into a multivariable model, psychological distress was independently associated with lower scores on all five scales, while regular psychedelic use was associated with higher scores on the PTG scales. See Fig. 2 for a graphical representation of the corrected means of the five PTG scores for never, occasional, and regular users of psychedelic drugs.

**Table 4:** Multivariable longitudinal analyses to assess associations between various stress-related factors and baseline psychedelic substance use and posttraumatic growth at the follow-ups (2 and 6 months later).

	Post-traumatic growth scores^b^									
	Relating to others		New possibilities		Personal strength		Spiritual change		Appreciation of life	
	B (SE)	p	B (SE)	p	B (SE)	p	B (SE)	p	B (SE)	p
Peritraumatic stress^a^	0.87 (0.35)	0.01	0.56 (0.27)	0.04	0.41 (0.23)	0.07	0.19 (0.10)	0.06	0.34 (0.18)	0.06
General severity index (BSI)	0.05 (0.03)	0.10	0.01 (0.03)	0.69	0.02 (0.02)	0.44	0.01 (0.01)	0.49	0.04 (0.02)	0.04
Psychological distress (GHQ)	− 0.24 (0.06)	** < 0.001**	− 0.23 (0.05)	** < 0.001**	− 0.24 (0.04)	** < 0.001**	− 0.09 (0.02)	** < 0.001**	− 0.11 (0.03)	** < 0.001**
Occasional use vs. never^a^	1.50 (0.70)	0.03	1.46 (0.53)	0.01	0.76 (0.50)	0.10	0.34 (0.19)	0.07	0.50 (0.35)	0.16
Regular use vs. never^a^	3.93 (0.81)	** < 0.001**	3.38 (0.56)	** < 0.001**	2.31 (0.51)	** < 0.001**	1.22 (0.22)	** < 0.001**	1.69 (0.38)	** < 0.001**

Generalized estimated equations were corrected for age, gender, language of questionnaire, and religion. Significant (Bonferroni-corrected) p-values ≤ 0.003 are represented bold.

^a^Only recorded at baseline; b) Only recorded at the follow-ups.

### Transcultural comparison

Supplementary Tables 1, 2 and 3 show the differences between the three languages in which the questionnaires were completed. At all three time points, there were significant differences between the English, Spanish, and Portuguese speakers in their rating of the information given by the media and politicians, their stress levels, BSI scores, and the use of psychedelic drugs. Results were heterogeneous, and no clear tendency can be observed.

## Discussion

In this manuscript, we explore the relationship between hallucinogen use and different variables, including mental health and well-being, peritraumatic stress, and PTG. This study was initiated during a global pandemic, when many countries were implementing confinement measures.

Results showed that lifetime use of hallucinogens was related to better scores on most of the measures and that regular users had lower scores on each of the psychopathology scales. Regular users of hallucinogens also had higher PTG scores than occasional or never users, even after the multivariate longitudinal analysis corrected for various psychological measures. Within the multivariate model, it can be observed that as scores for psychological distress (GHQ-12) increased, lower scores on PTG were obtained, indicating that as distress reaches higher levels, there is a decrease of growth associated with such distress. It should be noted that both psychological distress and regular use of hallucinogens were independently associated with PTG.

Some important questions related to clinical and public policy arise from our results. Contrary to the presumptions that justify hallucinogens being scheduled substances (i.e., causing harm to health and having a high abuse potential), our results show that they might offer some kind of protection of mental health when confronting highly stressful situations, like facing a pandemic where most of the world’s population is suffering unprecedented deaths and strict lockdowns. Another retrospective, transversal study (n = 5618), performed at the same time as our study, also found a relationship between lifetime use of hallucinogens and lower scores on dimensions linked to mental health impairment (state/trait anxiety, negative affect), as well as higher scores on dimensions linked to well-being and resilience (mainly positive affect, autonomy, social ties, and wellbeing)^34^. Other cohort studies have found positive relationships between lifetime use of hallucinogens and mental health^27,32,47,48^, and negative relationships between hallucinogenic drug use and suicidality^26,49^ and violence^50,51^. Additionally, there are numerous published manuscripts reporting the positive effects of hallucinogens on mental health in clinical settings (for reviews, see Goldberg et al.^52^; Luoma et al.^53^; and Yu et al.^54^) and in ceremonies where hallucinogens were administered^15,20,55–57^. Lastly, cross-sectional studies comparing regular hallucinogen users with non-users have found an absence of neuropsychiatric impairments in the former, with users reporting better scores than non-users on some measures^31,58–61^.

In a previous manuscript, we hypothesized that regular hallucinogen use could be a protective factor when confronting stressful situations^21^. The pandemic offered an unfortunate but appropriate scenario in which to test our hypothesis, and the results were in line with it: lifetime hallucinogen use was related longitudinally with positive outcomes in terms of most of the mental health measures. Moreover, regular use of hallucinogenic drugs was specifically associated with higher scores on the PTG scales (relating to others, new possibilities, personal strength, spiritual change, and appreciation of life). In relation to that, another manuscript focused on coping strategies and use of hallucinogens showed that regular users of these drugs are more prone to use adaptive coping strategies^62^. This is highly relevant in the context of the recently published research on the potential of MDMA for the treatment of PTSD^63^. PTG reflects an adaptive process of adjustment, and it is positively correlated with resiliency, meaning-making regarding the traumatic event, and functional relationships. Thus, PTG is an indicator of a holistic process of change following a traumatic event^54,64^. It is worth noting that we should be prevented of labeling the pandemic experience as a traumatic for everyone. Instead, the measure of PTG can be interpreted as an indicator of resilience, or positive-biased personal changes that can occur in generally significant experiences. There are only a couple of research reports that have assessed PTG in the context of MDMA-assisted psychotherapy^64,65^. Our current results add evidence to these emerging findings, extending from MDMA to the whole class of hallucinogenic drugs, suggesting that the effects of MDMA on PTG may be a non-specific feature of hallucinogenic drugs regarding the treatment of trauma.

The hallucinogenic experience produced by drugs like psilocybin and ayahuasca has been characterized as spiritual and/or mystical^66,67^. Commonly, the phenomenon so-called “ego dissolution” occurs^68^, and personal insights can emerge, providing subjects with new perspectives about themselves, their relationships, and the nature of reality^69^. These features tend to be conceived of as positive, leading to transformative and valuable experiences. However, psychedelic experiences can also be extremely stressful^70–74^. Despite this, it is common for users to continue taking these drugs after having experienced certain adverse effects, especially in the case of ayahuasca^75^. It is possible, therefore, that the willingness that users tend to show toward exposing themselves to highly challenging experiences is somehow related to their better PTG scores. In addition, this continuous exposure to challenging experiences could have provided personal resources to deal with pandemic stress, partially explaining the better scores on mental health and well-being they showed in different studies^34,62^. Previous research has shown different personality traits between hallucinogenic drug users and non-users, especially in the case of ayahuasca, as well as differences between users depending on the environment in which they live^31,76^. These differences may provide possible explanations for our findings. Taking into account the hierarchical order of psychological variables suggested by some authors^34^, we observe that personality domains influence on cognitive styles, which are responsible for interpreting and processing reality. These interpretations, in turn, affect emotions, and emotional states contribute to complex self-perceived processes that we conceptualize as well-being or resilience. Therefore, the ability of psychedelics to modulate personality traits, particularly openness and self-transcendence^35,75^, may be at the core of a "cascade" of subsequent psychological changes that eventually result in protective effects against various stressors, including pandemic-related stress. Further research in that regard is warranted.

Another remarkable result was the association between regular use of hallucinogens and lower psychopathology scores. The public image of hallucinogens has associated their use with madness and bizarre behaviors. In addition, hallucinogenic drugs have been used to mimic psychotic states^77^. Nevertheless, our results, in concordance with recent research on hallucinogens^78^, show that the use of these drugs is associated with less psychopathology and with functional behaviors. Many variables could be intertwined with these outcomes. For instance, in the case of the present study, regular users of hallucinogenic drugs also showed an enhanced detachment from the information given by politicians and the media, which has been repeatedly related to higher distress scores during the initial waves of the pandemic^79,80^. Remarkably, a recently published preclinical study suggested that the use of ayahuasca could have a prophylactic effect in front of depressive symptoms^81^. Thus, a similar mechanism might be responsible for the present findings.

Due to our social responsibility as scientists, we must emphasize the policy implications of these results. Most classical hallucinogens (LSD, psilocybin, DMT or MDMA) are in the Schedule I of the United Nations (UN) Convention on Psychotropic Drugs of 1971 for their severe health risks. Although drugs included in the Schedule I are only allowed to be used for medical and scientific purposes, their use in non-medical settings has remained highly prevalent in Western countries. Notably, scientists are asking for the reclassification of these substances^82,83^ given the emerging body of evidence, which the present study strengthens by showing the positive consequences of their use in both informal and experimental settings. A policy that it is not aligned with scientific evidence should be changed for the benefit of society. Good examples of this proposed change include local public policies like the one developed in Alberta (Canada), Oregon (USA) or in Catalonia, where the local government adopted a harm reduction approach regarding the extended use of ayahuasca^84^.

The most relevant limitation of this study was the high drop-out rate during the follow-ups of this study, a limitation that tends to occur in longitudinal studies. Although characteristics like age, gender, and marital status remained the same in each assessment, this drop-out rate might have affected some other characteristics of the sample, giving the longitudinal analyses limited reliability. For instance, the percentage of users of hallucinogenic drugs in the sample dropped considerably across the follow-ups. Nevertheless, in terms of the English version of the questionnaire, the majority of the participants in both follow-ups were users of hallucinogenic drugs, as they were possibly more motivated to answer a survey in which they were asked about their use of hallucinogens. One additional limitation of our study is the comparison of subjects between different language versions of the questionnaire (English, Portuguese, and Spanish), rather than comparing subjects between countries. The latter option would have been challenging due to our sample being provided by individuals from more than 80 countries. However, we opted to group Portuguese-speaking individuals from both Portugal and Brazil, as well as Spanish-speaking individuals from both Spain and South American countries. It is important to note that the reality of the pandemic may have varied substantially in these different regions. Lastly, self-report measures have inherent limitations and may be influenced by biases, such as individuals who already perceive consumption of psychedelic drugs positively. We employed validated assessment tools to minimize subjective interpretation and increase the reliability of our findings, but the results have to be interpreted with caution.

One of the main inspirations for the design of this study was the uncommon scenario of widespread social isolation during confinement measures. While this scenario provided an interesting object of study, it could also be seen as an extraneous variable, since we presume that confinement measures could have affected the number of people who answered the survey. At the baseline assessment, nearly all countries that participants were from were in strict lockdown (97% of the sample were confined), whereas at the second and third assessments, 47% and 85.5% of participants were confined, respectively. Especially during the third assessment, six months after the first (in October, 2020), most countries developed other less strict measures, avoiding complete confinement precisely due to the psychological consequences observed. However, the majority of our sample at this assessment reported being confined (85.5%, which is in line with the high score obtained on the GSI in the last follow-up, as reported in Supplementary Table 4, suggesting that confinement is closely linked with psychological distress). Thus, it is possible that people in confinement were more likely to take 20–30 min to answer an online survey, biasing the results of the study toward confined people.

## Conclusions

Users of hallucinogenic drugs scored better on various dimensions that were clearly affected by the recent COVID-19 pandemic, like psychopathology, wellbeing, and post-traumatic growth. This was even clearer in the case of regular users of these drugs. From these results, we can suggest that either these drugs exert certain effects on individuals that protect them against some of the distress of life-changing events, or people who use these drugs do so because they have traits that are related to the better management of stressful events. Overall, these findings have public policy implications that should be addressed. Further research should elucidate the role of hallucinogenic drugs in large-scale catastrophes, such as pandemics and wars.

### Supplementary Information

Supplementary Information.

## Data Availability

The raw data is available upon request to Dr. JC Bouso, corresponding author.
