# Supplementary material for: Longitudinal and transcultural assessment of the relationship between hallucinogens, well-being, and post-traumatic growth during the COVID-19 pandemic
Source: Sci Rep. 2023 Sep 11;13:14052. doi: 10.1038/s41598-023-41199-x (PMC10495368; doi:10.1038/s41598-023-41199-x)
Supplement: Supplementary file 1 — Supplementary Information. [file 41598_2023_41199_MOESM1_ESM.docx]

| **Supplemental Table 1: Comparing means and standard deviations of psychometric measures and psychedelic drug use at baseline among participants that filled out the questionnaire in Spanish, Portuguese and English** | | | | |
| --- | --- | --- | --- | --- |
| **Baseline** | **English (mean (SD))**  **N=671** | **Spanish (mean (SD))**  **N=1609** | **Portuguese (mean (SD))**  **N=691** | **p-value** |
| **Covid-related items rated on 1-10 scale** | | | | |
| Psychological wellbeing | 5.72 (2.36) | 5.87 (2.09) | 5.71 (2.01) | .14 |
| Home environment | 7.04 (2.28) | 7.28 (2.07) | 7.36 (2.22) | .02 |
| Information given by politicians | 3.83 (2.46) | 4.22 (2.42) | 3.93 (2.42) | **.001** ^b^ |
| Information given by media | 4.08 (2.25) | 4.18 (2.29) | 5.30 (2.39) | **<.001** ^cd^ |
| **Stress** | | | | |
| Psychological distress (GHQ) | 3.14 (3.33) | 2.67 (3.35) | 3.56 (3.60) | **<.001** ^d^ |
| **Brief Symptom Inventory scores** | | | | |
| Somatization | 56.22 (7.02) | 62.13 (10.92) | 56.29 (8.04) | **<.001** ^bd^ |
| Obsessive-compulsive | 63.74 (9.64) | 62.99 (9.70) | 64.52 (9.32) | **.002** ^d^ |
| Interpersonal sensitivity | 61.35 (9.90) | 60.22 (9.66) | 61.62 (9.76) | **.002** ^d^ |
| Depression | 63.09 (10.00) | 65.62 (10.85) | 63.48 (9.79) | **<.001** ^bd^ |
| Anxiety | 60.31 (8.99) | 63.45 (10.17) | 62.55 (8.91) | **<.001** ^bc^ |
| Hostility | 58.43 (8.09) | 56.93 (8.49) | 60.01 (7.84) | **<.001** ^bcd^ |
| Phobic anxiety | 61.51 (9.88) | 58.35 (10.07) | 59.57 (9.35) | **<.001** ^bc^ |
| Paranoid ideation | 58.15 (7.78) | 58.87 (8.08) | 60.81 (8.26) | **<.001** ^cd^ |
| Psychoticism | 57.94 (7.55) | 54.51 (5.96) | 59.97 (8.41) | **<.001** ^bcd^ |
| General severity index | 58.97 (6.23) | 58.75 (6.21) | 60.35 (6.70) | **<.001** ^cd^ |
| **Psychedelic drug use, N (%) ^a^** | | | | |
| Never | 243 (36) | 1052 (65) | 573 (83) | **<.001** ^bcd^ |
| Occasional | 205 (31) | 331 (21) | 70 (10) |  |
| Regular | 223 (33) | 226 (14) | 48 (7) |  |
| **Footnotes**: One-way ANOVA’s were ran to test differences between all questionnaires, and significant (Bonferroni-corrected) p-values ≤.003 are represented bold.  a) Only recorded at baseline; b) Significant difference between English and Spanish speakers; c) Significant difference between English and Portuguese speakers; d) Significant difference between Spanish and Portuguese speakers. | | | | |

| **Supplemental Table 2: Comparing means and standard deviations of psychometric measures and psychedelic drug use after 2 months among participants that filled out the questionnaire in Spanish, Portuguese and English** | | | | |
| --- | --- | --- | --- | --- |
| **After 2 months** | **English (mean (SD))**  **N=228** | **Spanish (mean (SD))**  **N=586** | **Portuguese (mean (SD))**  **N=210** | **p-value** |
| **Covid-related items rated on 1-10 scale** | | | | |
| Psychological wellbeing | 6.51 (2.18) | 6.75 (1.86) | 6.40 (1.83) | .04 |
| Home environment | 7.27 (1.88) | 7.45 (2.00) | 7.43 (2.09) | .52 |
| Information given by politicians | 3.92 (2.58) | 4.54 (2.51) | 2.99 (2.27) | **<.001** ^bcd^ |
| Information given by media | 4.19 (2.41) | 4.15 (2.28) | 5.61 (2.37) | **<.001** ^cd^ |
| **Stress** | | | | |
| Psychological distress (GHQ) | 4.86 (3.48) | 3.62 (3.69) | 4.15 (3.86) | **<.001** ^b^ |
| **Brief Symptom Inventory scores** | | | | |
| Somatization | 55.46 (6.03) | 58.38 (7.37) | 56.91 (6.65) | **<.001** ^b^ |
| Obsessive-compulsive | 61.24 (8.47) | 60.24 (8.62) | 64.10 (8.96) | **<.001** ^cd^ |
| Interpersonal sensitivity | 59.51 (8.43) | 59.38 (8.97) | 62.19 (9.26) | **<.001** ^cd^ |
| Depression | 60.31 (8.65) | 59.02 (7.35) | 62.44 (9.34) | **<.001** ^d^ |
| Anxiety | 57.69 (7.27) | 55.25 (5.56) | 60.79 (8.18) | **<.001** ^bcd^ |
| Hostility | 56.35 (6.38) | 57.05 (6.56) | 59.16 (6.60) | **<.001** ^cd^ |
| Phobic anxiety | 59.20 (8.97) | 56.98 (6.31) | 59.25 (8.76) | **<.001** ^bd^ |
| Paranoid ideation | 57.58 (6.62) | 55.23 (4.76) | 59.91 (8.48) | **<.001** ^bcd^ |
| Psychoticism | 56.02 (5.98) | 56.15 (6.32) | 58.87 (7.05) | **<.001** ^cd^ |
| General severity index | 58.91 (6.13) | 57.36 (6.34) | 60.55 (6.58) | **<.001** ^bd^ |
| **Posttraumatic growth scores ^a^** | | | | |
| Relating to others | 10.17 (8.38) | 10.98 (8.84) | 8.69 (7.82) | .004 ^d^ |
| New possibilities | 7.83 (6.45) | 8.19 (6.49) | 8.53 (6.42) | .52 |
| Personal strength | 6.24 (5.39) | 7.11 (5.79) | 6.17 (5.46) | .04 |
| Spiritual change | 2.08 (2.24) | 2.51 (2.66) | 2.44 (2.74) | .11 |
| Appreciation of life | 5.63 (4.06) | 6.21 (4.30) | 6.16 (4.19) | .20 |
| **Psychedelic drug use** | | | | |
| Non-user | 91 (40) | 442 (75) | 192 (91) | <.**001** ^bcd^ |
| User | 137 (60) | 144 (25) | 18 (9) |  |
| **Footnotes**: One-way ANOVA’s were ran to test differences between all questionnaires, and significant (Bonferroni-corrected) p-values ≤.003 are represented bold.  a) Only recorded at follow-ups; b) Significant difference between English and Spanish speakers; c) Significant difference between English and Portuguese speakers; d) Significant difference between Spanish and Portuguese speakers. | | | | |

| **Supplemental Table 3: Comparing means and standard deviations of psychometric measures and psychedelic drug use after 6 months among participants that filled out the questionnaire in Spanish, Portuguese and English** | | | | |
| --- | --- | --- | --- | --- |
| **After 6 months** | **English (mean (SD))**  **N=16** | **Spanish (mean (SD))**  **N=305** | **Portuguese (mean (SD))**  **N=134** | **p-value** |
| **Covid-related items rated on 1-10 scale** | | | | |
| Psychological wellbeing | 7.25 (1.88) | 6.70 (1.87) | 6.51 (1.97) | .30 |
| Home environment | 7.81 (1.76) | 7.45 (1.88) | 7.31 (2.12) | .56 |
| Information given by politicians | 4.31 (2.89) | 4.07 (2.36) | 3.14 (2.28) | **.001** ^d^ |
| Information given by media | 4.44 (2.28) | 3.81 (2.30) | 5.87 (2.18) | **<.001** ^cd^ |
| **Stress** | | | | |
| Psychological distress (GHQ) | 2.38 (3.10) | 3.36 (3.89) | 3.70 (3.77) | .37 |
| **Brief Symptom Inventory scores** | | | | |
| Somatization | 56.88 (8.98) | 58.72 (7.53) | 56.34 (6.53) | .01 ^d^ |
| Obsessive-compulsive | 58.23 (9.48) | 59.96 (8.95) | 63.96 (9.66) | <.**001** ^d^ |
| Interpersonal sensitivity | 58.59 (10.37) | 59.30 (9.40) | 61.81 (10.28) | .04 |
| Depression | 58.54 (9.73) | 59.32 (7.66) | 62.38 (9.56) | **.002** ^d^ |
| Anxiety | 54.69 (8.59) | 55.08 (5.65) | 60.12 (8.58) | **<.001** ^cd^ |
| Hostility | 55.38 (9.20) | 57.12 (7.22) | 59.10 (7.60) | .02 |
| Phobic anxiety | 55.25 (7.23) | 56.66 (6.78) | 59.69 (9.19) | **<.001** ^d^ |
| Paranoid ideation | 56.38 (7.60) | 55.21 (4.46) | 60.12 (8.47) | **<.001** ^d^ |
| Psychoticism | 57.00 (9.41) | 56.08 (6.59) | 57.91 (7.25) | .04 |
| General severity index | 58.02 (8.03) | 58.80 (7.60) | 60.09 (7.60) | **.**22 |
| **Posttraumatic growth scores ^a^** | | | | |
| Relating to others | 9.19 (7.31) | 10.00 (8.71) | 8.20 (7.72) | **.**12 |
| New possibilities | 9.31 (5.56) | 8.00 (6.65) | 8.44 (6.31) | .63 |
| Personal strength | 6.94 (5.56) | 6.83 (5.88) | 6.14 (5.79) | .51 |
| Spiritual change | 2.50 (2.25) | 2.40 (2.67) | 2.48 (2.65) | .96 |
| Appreciation of life | 6.56 (3.72) | 5.93 (4.26) | 6.49 (4.00) | .40 |
| **Psychedelic drug use** | | | | |
| Non-user | 6 (38) | 224 (73) | 119 (89) | <.**001** ^bcd^ |
| User | 10 (63) | 81 (27) | 15 (11) |  |
| **Footnotes**: One-way ANOVA’s were ran to test differences between all questionnaires, and significant (Bonferroni-corrected) p-values ≤.003 are represented bold.  a) Only recorded at follow-ups; b) Significant difference between English and Spanish speakers; c) Significant difference between English and Portuguese speakers; d) Significant difference between Spanish and Portuguese speakers. | | | | |

| **Supplemental table 4: Amount of persons above the cut-off scores for each BSI subscale and the GSI at each time point** | | | |
| --- | --- | --- | --- |
|  | T-score above 70 | | |
| **BSI scores** | **Baseline N=2970** | **After 2 months N=1024** | **After 6 months N=455** |
| Somatization | 441 (14.8) | 63 (6.2) | 31 (6.8) |
| Obsessive-compulsive | 688 (23.2) | 154 (15.0) | 69 (15.2) |
| Interpersonal sensitivity | 482 (16.2) | 122 (11.9) | 64 (14.1) |
| Depression | 801 (27.0) | 118 (11.5) | 49 (10.8) |
| Anxiety | 587 (19.8) | 60 (5.9) | 24 (5.3) |
| Hostility | 259 (8.7) | 47 (4.6) | 28 (6.2) |
| Phobic anxiety | 442 (14.9) | 79 (7.7) | 36 (7.9) |
| Paranoid ideation | 292 (9.8) | 33 (3.2) | 17 (0.6) |
| Psychoticism | 181 (6.1) | 38 (3.7) | 26 (5.7) |
|  | T-score above 63 | | |
| General severity index | 766 (25.8) | 227 (22.2) | 119 (26.2) |
| **Footnotes**: Higher scores represent more severe symptoms, and persons above the cut-off scores are seen as clinical cases. | | | |
